# The HIV-1 Integrase α4-Helix Involved in LTR-DNA Recognition Is also a Highly Antigenic Peptide Element

**DOI:** 10.1371/journal.pone.0016001

**Published:** 2010-12-30

**Authors:** Sandy Azzi, Vincent Parissi, Richard G. Maroun, Pierre Eid, Olivier Mauffret, Serge Fermandjian

**Affiliations:** 1 LBPA, ENS de Cachan, CNRS, Cachan, France; 2 Unité Inserm 1014, Régulation de la survie cellulaire et des allogreffes, Université Paris XI, Villejuif, France; 3 CNRS UMR 5234, Laboratoire de Microbiologie Cellulaire et Moléculaire et Pathogénicité, Université de Bordeaux II, Bordeaux, France; 4 Département des Sciences de la Vie et de la Terre, Faculté desSciences, Université Saint-Joseph, CST-Mar Roukoz, Beirut, Lebanon; Institut Pasteur, France

## Abstract

Monoclonal antibodies (MAbas) constitute remarkable tools to analyze the relationship between the structure and the function of a protein. By immunizing a mouse with a 29mer peptide (K159) formed by residues 147 to 175 of the HIV-1 integrase (IN), we obtained a monoclonal antibody (MAba4) recognizing an epitope lying in the N-terminal portion of K159 (residues 147–166 of IN). The boundaries of the epitope were determined in ELISA assays using peptide truncation and amino acid substitutions. The epitope in K159 or as a free peptide (pep-a4) was mostly a random coil in solution, while in the CCD (catalytic core domain) crystal, the homologous segment displayed an amphipathic helix structure (α4-helix) at the protein surface. Despite this conformational difference, a strong antigenic crossreactivity was observed between pep-a4 and the protein segment, as well as K156, a stabilized analogue of pep-a4 constrained into helix by seven helicogenic mutations, most of them involving hydrophobic residues. We concluded that the epitope is freely accessible to the antibody inside the protein and that its recognition by the antibody is not influenced by the conformation of its backbone and the chemistry of amino acids submitted to helicogenic mutations. In contrast, the AA →Glu mutations of the hydrophilic residues Gln148, Lys156 and Lys159, known for their interactions with LTRs (long terminal repeats) and inhibitors (5

CITEP, for instance), significantly impaired the binding of K156 to the antibody. Moreover, we found that in competition ELISAs, the processed and unprocessed LTR oligonucleotides interfered with the binding of MAba4 to IN and K156, confirming that the IN α4-helix uses common residues to interact with the DNA target and the MAba4 antibody. This also explains why, in our standard *in vitro* concerted integration assays, MAba4 strongly impaired the IN enzymatic activity.

## Introduction

HIV-1 replication requires the use of three enzymes encoded by the Gag/Pol gene: reverse transcriptase, protease and integrase (IN) [1], [2]. After virus entry into host immune cells, reverse transcriptase converts the HIV-1 RNA into DNA. Then IN carries out integration of viral DNA into the host chromosome through a two-step process: 3′ processing and strand transfer. Initially, a dinucleotide GT is excised from the 3′ ends (transferred strand) of nascent DNA in the cytoplasm. A multi-component pre-integration complex, including the processed viral DNA and IN, is chaperoned into the nucleus. Here occurs the covalent insertion of HIV-1 DNA into the host chromosome [3], [4], [5].

The HIV-1 IN as the other retroviral INs comprises three distinct domains [6], [7]: the Nterminal domain (NTD), the C-terminal domain (CTD) and the core catalytic domain (CCD). NTD (residues 1-50) exhibits a three helix bundle organization with a helix-turn-helix motif bound to Zn2+ [8]. CTD (residues 213–288) contains a structure similar the SH3 motif involved in protein-protein interactions and is rich in Lys and Arg residues distributed on β-strands [9], [10]. The central CCD (residues 51–212) is formed of 5 β-strands and 6 α helices and harbors the conserved catalytic triad of acidic residues D, D, E -that binds either one or two divalent ions (i.e. Mg2+ or Mn2+) - embedded in an RNase fold [11], [12], [13], [14], [15], [16], [17], [18], [19].

The three domains taken separately or coupled in two-domain fragments (CCD-CTD and NTD-CCD) form a dimer [20], although the tetramer emerges as the functional association [21], [22], [23], [24], [25]. Both the 3′-processing and the DNA joining reactions have been reproduced in *in vitro* assays with the recombinant IN and duplex oligonucleotides mimicking the U3/U5 LTR extremity with a processing site CA↓GT at the 3′-end of the transferred strand. Such 17 to 21 base-pair oligonucleotides behave *in vitro* as both DNA donor and DNA acceptor. A large number of mutations or modifications have suggested the key role of the six outermost base-pairs for binding of IN to virus DNA [1], [12]. This has been recently confirmed by the crystal structure of the Protoype Foamy Virus (PFV) in complex with a 3′-processed cognate LTR DNA [25]. Analysis of the crystal structure of the above complex has further highlighted the key role held by the amphipatic α4-helix of CCD in the recognition of virus.

**Figure 1:**
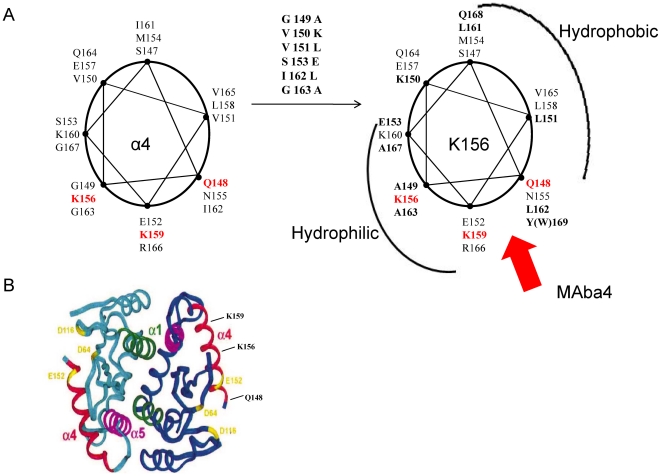
Structural properties of the HIV-1 IN α4-helix. A: In the various crystal structures of the IN CCD, the α4-helix is located at the protein surface with its hydrophobic residues turned towards the interior of the protein and the polar residues Gln148, Lys156 and Lys159 directed outwards from the protein, prone to interact with the antibody and the DNA. B: The α-4 helix and its structural analogue K156 are represented as a helical wheel, with the N-terminal residue S147 positionned at the top on the circle. Most of substitutions converting pep-a4 into the analogue K156 are hydrophobics and rather isosterics (Gly→Ala, Val→Leu, Ile→Leu…). The global effect of mutations is an improvement of the helicity [26]. Note that residues Gln148, Lys156, Lys159 are aligned on a narrow ridge on the hydrophilic/charged surface of the helix.

DNA, this lending credence to our previous results [26], [27], [28], [29]. Actually, the strongest binding determinants of the α4-helix are: its global accessibility at the CCD surface, and, especially, the large exposition to solvent of the polar/charged side chains of residues as for instance Gln148, Lys156 and Lys159 (Fig. 1-A). Implication of these residues in binding of IN to virus DNA and strand transfer inhibitors [1] has been shown by mutagenesis [12], [30], [31], [32], chemical modifications [30], [33], [34], [35], [36], [37], [38] and spectroscopy methods in solution [26], as well as analysis of the crystal structure of the 5CITEP-CCD complex [39] and drug resistance mutations [40], [41], [42], [43], [44].

To go thoroughly into above points and related questions, we prepared monoclonal antibodies against a synthetic peptide named K159 reproducing the sequence 147–175 of the HIV-1 IN [45]. Within the CCD crystal structure the corresponding segment carries the α4- helix in its N-terminal portion (residues from about 150 to 166), a loop in its center (residues 166–171) and the beginning of the α5-helix in its C-terminal portion (residues 172–175) [15], [16], [19]. Indeed, the whole K159 peptide appears highly antigenic. About 10 year ago we had already characterized a functional epitope in its C-terminal portion [46]. Once purified to the stage of monospecific the corresponding polyclonal antibodies were able to recognize with high affinity the isolated C-terminal portion, the entire K159, CCD and the full-length enzyme. They were also strong antagonists of the IN enzymatic activity [46]. Here, an optimal epitope was found in the N-terminal portion (residues 147–166), using peptide truncation and ELISA assays. The monoclonal antibody prepared against K159, MAba4, recognizes with almost similar affinity the epitope in a random coil structure (peptide-a4 either isolated or include in K159) or in a helical conformation (within either IN or the peptide K156, an analogue of the peptide-a4 stabilized into a helical conformation thanks to seven helicogenic substitutions performed in selected positions) (Fig. 1B) [26]. These substitutions while efficient to stabilize the conformation do not partipate to the complex formation. In contrast, those substitutions performed on residues Gln148, Lys156 and Lys159 [26], [47] which contribute to the binding of IN to the virus DNA and also the 5CITEP inhibitor [39], have deleterious effects on the binding to MAba4. The antibody incubated with IN inhibits its enzymatic activity in *in vitro* concerted integration assays [22], [48], [49]. Use of LTR DNAs in competition ELISA assays reveals that these latter compete with the antibody for the binding to IN and K156. We also noted that the unprocessed version of LTR DNA is a better competitor than the processed version for the binding of MAba4 to IN. These results actually correlate with our previous observation showing that IN had a higher affinity for unprocessed DNA than for processed DNA, thus highlighting the role of the GT3′ dinucleotide in the stabilization of the IN-DNA complex.

## Materials and Methods

All animal work has been conducted according to relevant national and international guidelines. All animal care and procedures were in accordance with institutional guidelines and European regulations. Animal work has been done in INSERM agreated laboratory and production of antibodies was for the only aim of fundamental research. Therefore, no ethic committee approval was needed.

Agrément N°: C94-076-32 du 26 november 2009

CNRS-SEAT-UPS44

7 rue Gue Môquet- Bâtiment G

94800 VILLEJUIF

### Peptides and samples

**Figure 2:**
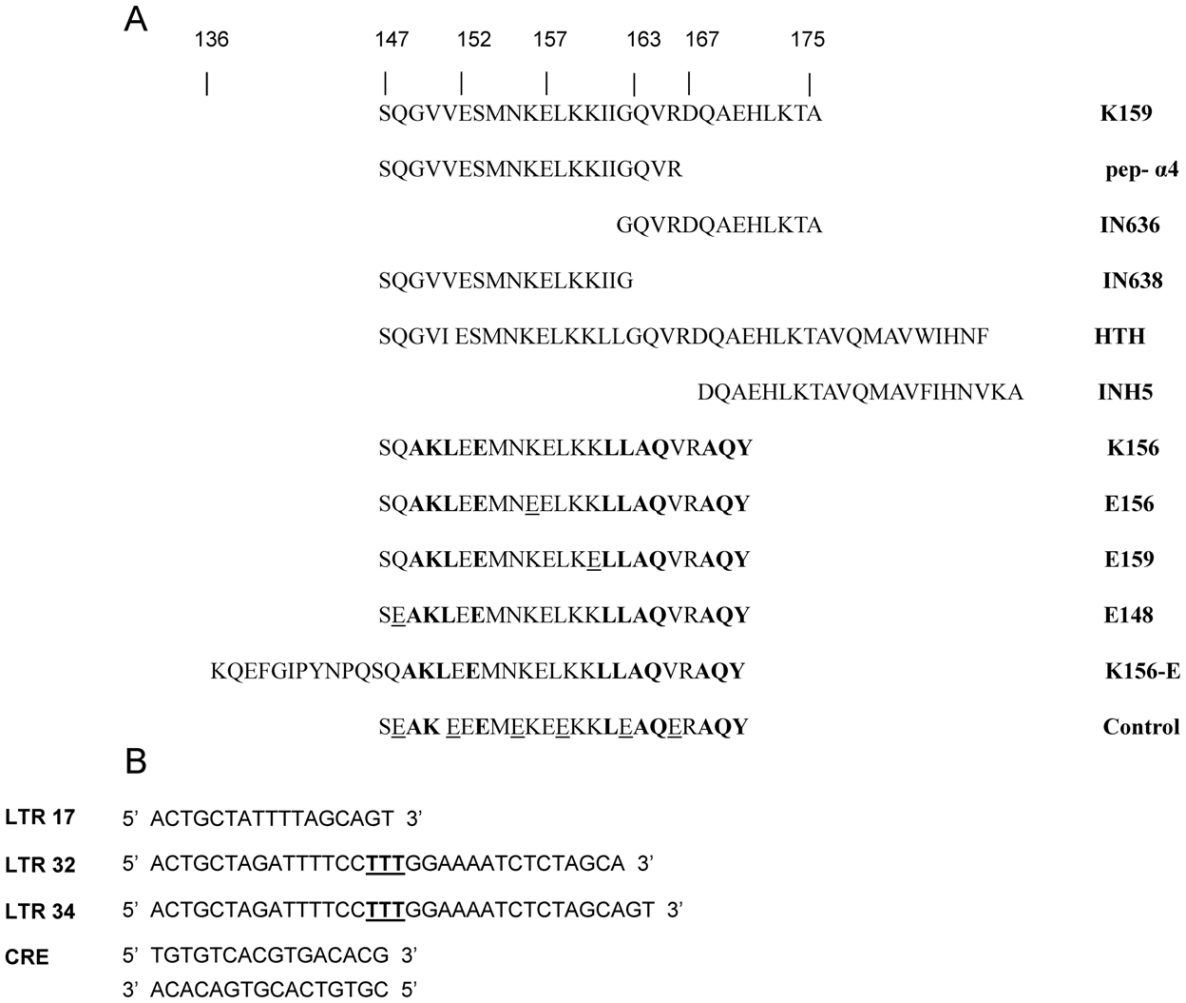
Peptides and oligonucleotides used to prepare and study the epitope. A: The 29-mer peptide K159 (residues 147–175 in IN of HXB2) was used as immunizing peptide [45], [46]. To delimit the epitope and analyze the properties we used several other peptides: pep-a4 reproducing the α4-helix sequence; IN636, a C-terminal fragment of K159 that has shown epitope properties in a previous work [46]; IN638, a N-terminal fragment of K159; INH5, a strong inhibitor of IN [46], that includes a loop region (residues 167–171) and the beginning of the α5-helix (residues 172–187); K156, a structural analogue of pep-a4, that is constrained into helix through seven helicogenic substitutions [26]; HTH (α4-helix-loop-α5-helix) [27]; E156, E159 and E148 (structural analogues of K156 in which residues Lys156, Lys159 and Gln148 has been respectively replaced by Glu); K156-E, the elongated K156 peptide ([136–146]-K156); and the peptide control that derives from K156 from six AA→Glu mutations. B: Once folded into hairpin structure around the central trinucleotide **TTT**, the three LTRoligonucleotides mimic the U5 LTR extremity. LTR17 carries a 7 pb stem corresponding to the most important region for IN binding. LTR34 carries a 17 pb stem, a minimum DNA size for successful reaction of integration *in vitro*. It represents an unprocessed version of U5 LTR, while LTR32 corresponds to the processed version with its 15 bp and its 5′CA hanging dipeptide. The DNA duplex CRE (cAMP responsive element) is used as a control.

The peptides used in the present work either reproduce or derive from a segment contained in the CCD of IN (Fig. 2A). Most of them have been already used in previous studies dealing with the identification of polyclonal antibodies against IN [46] and the binding of IN to LTR DNAs [26]. These were prepared according to the Fmoc [N-(9-fluorenyl) methoxycarbonyl] procedure and purified with reverse-phase high pressure liquid chromatography. The molecular mass and purity of each peptide was confirmed by electrospray ionization mass spectrometry. The peptide concentration was generally determined from the UV signal of Tyr or Trp purposely added at the peptide C-terminus, using molar absorption coefficient of 1197 M-1cm-1 at 280 nm (Tyr) and 5600 M-1cm-1 (Trp).

### DNA samples

The oligonucleotides were purchased from Eurogentec (Fig. 2B). The three LTRs of oligonucleotides were designed to adopt a hairpin structure. Compared with linear duplexes, they present a higher stability at the low concentrations used in ELISA experiments. LTR34 and LTR17 contain a 17-bp stem and a 7-bp stem, respectively, corresponding to the outermost domains of unprocessed U5 LTR, while LTR32 represents the 3′processed version of U5 LTR. The loop is formed around the three thymine sequence (TTT) inserted at the oligonucleotide centre. The oligonucleotide duplex CRE (cAMP responsive element) was used as a control.

### HIV-1 IN

Standard purification was performed essentially as previously described [50]. The soluble fraction containing the HIV-1 IN obtained from JSC 310 (IN) expressing IN protein was loaded on a Hitrap butyl-sepharose 4B column (1 ml, Pharmacia-LKB), washed with LSC buffer (50 mM HEPES pH 7.6, 0.2 M NaCl, 0.1 mM EDTA, 1 mM DTT, 7 mM CHAPS, 10% glycerol) and equilibrated with 5 volumes HSC buffer (50 mM HEPES pH 7.6, 0.2 M NaCl, 1 M ammonium sulfate, 0.1 mM EDTA, 1 mM DTT, 7 mM CHAPS). Proteins were eluted by a decreasing ammonium sulfate gradient (1 to 0 M). Fractions containing IN activity were pooled and 7 mM CHAPS was added. Pooled fractions were 1/3 diluted with 50 mM HEPES pH 7.6, 0.1 mM EDTA, 1 mM DTT, 10% glycerol, 7 mM CHAPS and loaded on a Hitrap Heparine Sepharose CL-4B column (1 ml, Pharmacia-LKB), washed with 5 volumes HS buffer (50 mM HEPES pH 7.6, 1 M NaCl, 0.1 mM EDTA, 1 mM DTT, 10% glycerol, 7 mM CHAPS) and equilibrated with a linear NaCl gradient (0 to 1 M NaCl). Fractions containing IN activity (eluted at 300 mM NaCl concentration) were pooled and concentrated or not by ultrafiltration (Centricon Millipore), followed by addition of 7 mM CHAPS. ZnSO4, 50 µM was added in the stock fraction. Purified IN (10 to 50 µM) was kept at −80°C (the NaCl concentration of the stock was 300 mM). Proteins were analyzed by electrophoresis in a 12% SDS-PAGE and western blotting using a polyclonal anti-IN antibody (Invitrogen).

### Antibody production and purification

Seven different monoclonal antibodies were selected according to a standard procedure. Among these two antibodies bound the peptide K159 at its N-terminal and C-terminal extremities with high affinity. We selected MAba4 recognizing the N-terminal extremity for the present study. Hybridoma cells were produced by fusing myeloma cells with the spleen cells from a C57 Black mouse that was immunized with the peptide K159, homologous to residues 147–175 of IN from HXB2D viral strain (Fig. 2A). This sequence is highly conserved within all HIV-1 strains and presents a very good homology with other retroviral integrases and tranposases [11], [51]. We had previously used this peptide to raise polyclonal antibodies in the rabbit [46]. Here, the peptide was coupled to Keyhole Limpet Hemocyanin (KLH-Pierce) with benzidine and injected to mice (200 µg per injection). The first injection was given subcutaneously in complete Freund's adjuvant. A booster injection was given on day 20 that is nearly three weeks after the first injection. The mice were bled on day 40. Cells isolated from the spleen were fused with myeloma cells (X63-Ag8) using PEG (polyethylene glycol).

Fused cells were then selected in a HAT (hypoxanthine aminopterin thymidine) medium. Hybridomas were harvested, diluted, and clones were grown from single parent cells on microtiter wells. MAba4 was purified from the corresponding hybridoma cell culture supernatant. An enzyme-linked immunosorbent assay (ELISA) was performed against K159 and HIV-1 IN and permitted to calculate the antisera titers. The inability of the antibody to bind to protein G in affinity column shows that this antibody belongs to IgA type. Purification of MAba4 was performed on a sepharose 4B column coupled with anti-IgA and controlled by a migration on a SDS-PAGE. Antibody concentration was determined by spectrophotometry at 280 nm.

### Immunoassays

We used simple ELISA assays to locate the antibody-binding region within the K159 peptide. The use of truncated K159 peptides and other related peptides (Fig. 2A) provided the amino acid positions of the epitope boundaries. Competition ELISAs were also used in order to assess the ability of IN target DNAs to move the antibody from its complex with IN or K156 (Fig. 2B). 5 µg of antigen (synthetic peptide or IN) were incubated in the wells of the reaction's plate. The plate was then incubated at 4°C overnight, and saturated with a 0.5% solution of bovine gelatin for 30 minutes at 37°C. 50 µl of MAb at 20 µg/ml were then added to each well and the mixture was incubated for 1 hour at 37°C. Wells were washed 5 times with the washing solution (PBS 1X +0.1% Tween 20) and 150 µL secondary Abs labeled with peroxidase and were distributed to sink to the dilution of 1/1000 in dilute solution (PBS 1X +0.1% Tween 20+0.5% gelatin). The plate was incubated 1 hour at 37°C to allow the binding of the secondary antibody. Revelation was performed by enzymatic reaction after washing sink with wash solution. 50 µL solution revelation (5 mg Ortho-Phenylenediamine: OPD) dissolved in 10 mL development (0.1 M citric acid +0.1 M trisodium citrate, pH = 6), and 10 µL H2O2 at 30% were added to each well and the plate was placed 20 to 30 minutes in the dark. The reaction was stopped with 25 µL hydrochloric acid 10^3^ M and reading was performed at 450 nm. U5/U3 LTR DNAs are targets of IN and K156, the stabilized analogue of pep-a4 mimicking the α4-helix of IN. To understand whether these oligonucleotides compete with MAba4 for the binding to IN or K156, competition ELISAs were performed with LTR17, LTR32, and LTR34, taking CRE (cAMP responsive element) and a mouse IgA antibody (Sigma, clone TEPC15) as controls. 5 µg of antigen (IN or K156) and 50 ng of DNA were preincubated at 37°C for 1 hour, and wells were coated with the IN-DNA complex and incubated overnight at 4°C.

### IN activity assays

Standard concerted integration reactions were performed as described previously [52], except that no cellular proteins were added. Briefly, purified HIV-1 IN (1 pmole, 50 nM) was preincubated with both the 5′-end-labeled donor DNA (10 ng) containing the 3′-processed U3 and U5 LTR sequences and the target DNA plasmid pBSK+ (100 ng) at 0°C for 20 min in a total volume of 5 µl. Then the reaction mixture (20 mM HEPES, pH 7.5; 10 mM DTT; 7.5 mM MgCl2; 10% DMSO; 8% PEG) was added and the reaction proceeded for 90 min. In all the reactions the final NaCl concentration was adjusted at 30 mM. Incubation was stopped by adding a phenol/isoamyl alcohol/chloroform mix (24/1/25 v/v/v). The aqueous phase was loaded on a vertical 1% agarose gel in the presence of 1% bromophenol blue and 1 mM EDTA. After separation of the products, the gel was treated with 5% TCA for 20 min, dried and autoradiographed. All IN activities were quantified by scanning of the bands after gel electrophoresis and autoradiography using the Image J software. Both target and donor plasmids were kind gifts from Dr. Karen Moreau (Université Claude Bernard-Lyon I, France).

The target corresponds to the plasmid pBSK+ (Stratagene, La Jolla, California) carrying the zeocin resistance encoding gene. The 294 bp pre-processed donor substrate was obtained as described previously [22] and contains after cleavage by NdeI the supF tRNA gene flanked by two pre-cleaved extremities mimicking the 3′-processed U3 and U5 LTR sequences. The unprocessed donor was generated by cloning a donor containing ScaI ends into a PGem-T vector (Promega) as previously described [53]. The PGem-T-SupFScaI resulting vector was cleaved by ScaI and the substrate fragment was purified.

## Results

### Epitope Mapping

An antigenicity profile prediction showed that the 147–175 sequence was more antigenic compared with the other IN sequences [54]. Moreover, the N-terminal portion has been shown high antigenic in the Los Alamos database (http://www.hiv.lanl.gov/content/immunology).

**Figure 3:**
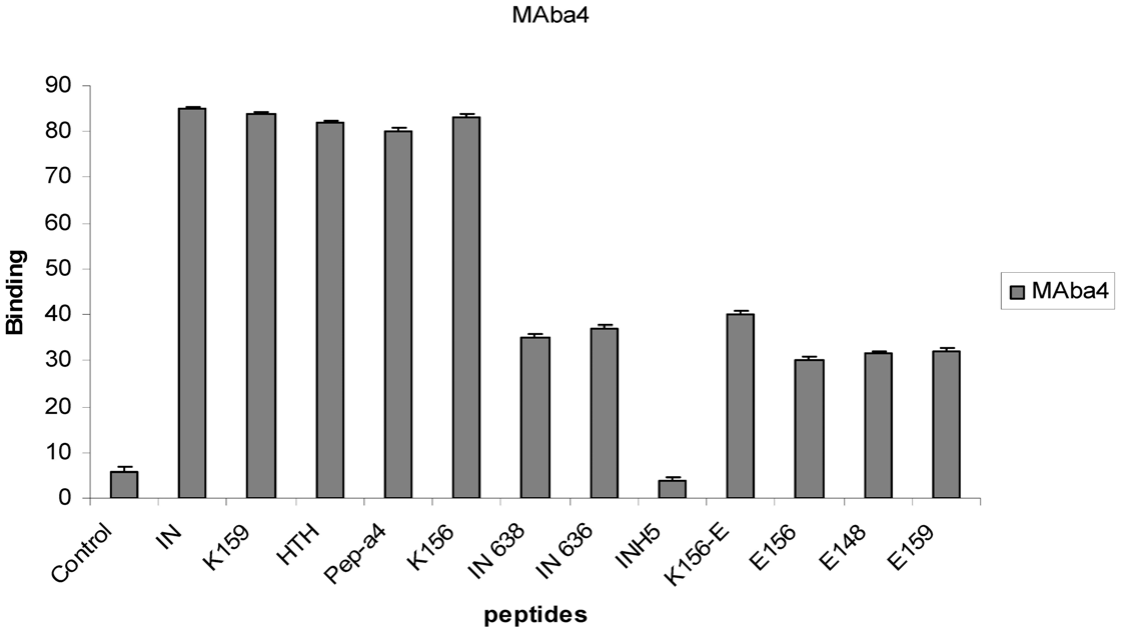
Identification of the epitope and residues that bind MAba4. The histogram represents the ELISA results provided by the binding of the MAba4 to the immunizing peptide K159, shorter and longer peptides, analogues and IN. ELISA absorbances with its target molecule are represented as a percentage of the mean absorbance for 12 experiments. The binding of MAba4 to peptide targets is expressed as the report of its absorbance to the control peptide. Panel values are the mean ± standard deviation (error bars) of three independent experiments.

The binding of MAba4 to IN, the antigenic peptide K159, and other peptide fragments (Fig. 2A) was studied by ELISA (Fig. 3). In these experiments, the signal intensity correlates to the ability of MAba4 to bind peptides and the IN protein. Signal for maximum interaction was observed with IN and K159, HTH, K156 and pep-a4. The observation of a same binding intensity for IN, pep-a4, K159 and HTH already suggests that the epitope coincides with pepa4 and is freely accessible to the antibody in the enzyme. The important loss of binding with the shorter IN636 peptide confirms that the epitope covers the all pep-a4 length. The binding conservation with INH5 further shows that the loop in between the α4- and the α5- helices is not necessary to recognition. However, comparison of pep-a4 with IN638 suggests that residues Val and Arg at the C-terminal end of the α4-helix, just before the loop, could participate to the interaction. Due to the high conservation of the 147–175 sequence within the intergrases of all HIV-1 strains, we can expect a good affinity binding of MAba4 also with these enzymes [11].

### Impact of sequence and conformation on recognition

We know from our previous CD experiments that the antigenic peptide K159 displays a high ratio of disordered secondary structure in aqueous solution; which is also the case of pep-a4 taken in isolation [26], [45], [55]. We have seen above that pep-a4 and K159 were recognized with a same high intensity by MAba4 (Fig. 3). Actually, it is also the case of the structural analogue of pep-4, K156, bearing seven amino acid substitutions (Fig. 1A). Most of these substitutions are helicogenic and concern hydrophobic residues which are buried in the CCD protein (Fig. 1B) [27]. These mutations restore the helix secondary structure, so that the resulting analogue K156 now mimics the α4-helix conformation displayed by the protein in the CCD crystal structure. Overall, the inability of MAba4 to discriminate between a stable and an unstable α4-helix reveals that the antibody binding is not sensitive to the epitope conformation. Moreover, the antibody is not measurably sensitive to the size and chemical nature of amino acid side chains, mostly located in the hydrophobic surface of the helix. In contrast, mutations performed in the hydrophilic surface of the helix show that the recognition is sensitive to the change of polar side chains exposed in this surface (Fig. 1A). The X→Glu replacements in positions 156 (Lys), 159 (Lys) and 148 (Gln) of K156 significantly impair the antibody recognition, each mutation dereasing three times the binding intensity. In fact these residues exert a key role in DNA cleavage and binding by IN [1], [26]; they also participate to the capture of the 5CITEP inhibitor by the IN [39]. Moreover, the position 148 (Glu) of IN is a hotspot for mutations conferring drug resistance, especially those induced by raltegravir [56].

Results obtained with the longer peptide K156-E (Fig. 2A) shed light on the question of the epitope/α4-helix accessibility to antibody and the possible masking effect of the well known flexible loop (residues 140–147) before the α4-helix. The weaker binding signal of K156-E compared with K156 suggests that the peptide moiety (residues 136–146) containing the loop hinders the recognition of the epitope by the antibody. Note that in the different crystal CCD structures so far reported, the loop extends beyond the area of the α4-helix. However, in the complex of the PFV (prototype foamy virus) IN- very similar to the HIV-1 IN- with its target DNA, the loop slightly folds back on the α4-helix and participate to strong contacts with the DNA. This binding of the loop (actually to the processed DNA) could somewhat prevent a near adjustment of the α4-helix to the DNA [25] concerning the binding of K156-E to the antibody, it could be also that the flexible loop folds back on the helix and, thus, interferes with the antibody recognition. The main victim of this particular folding could be residue Gln148 located at the loop-helix junction.

In the HTH motif the hydrophobic residues belonging to the amphipathic α4-helix participate to stabilizing interactions with the α5-helix, while the three polar/charged residues Gln148, Lys156 and Lys159 are exposed to solvent, free to contact any binding partner [27], [57]. Therefore, conservation of a high intensity binding with HTH, similar to pep-a4, is not so surprising.

### MAba4 hinders the binding of IN to DNA

**Figure 4:**
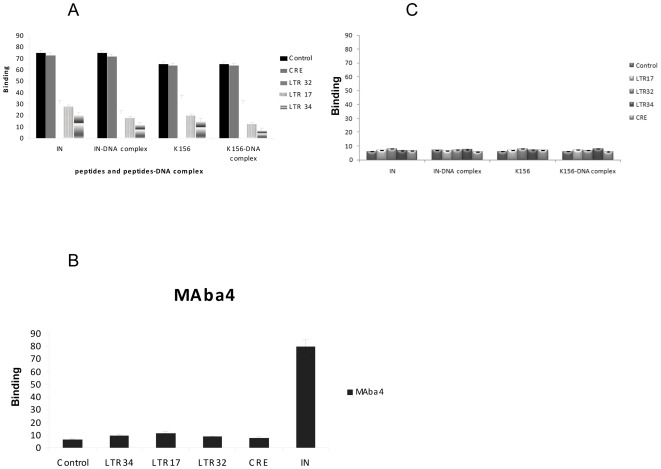
Inhibition of binding of MAba4 to IN and K156 by DNA fragments. A: Histogram representation of competition ELISA results for the competitive binding between DNAs and MAba4 to IN and K156. Wells were coated either with IN and K156 or with the complexes IN-DNA and K156-DNA. IN or K156 where incubated either with DNA or added to the antibody- complex. From left to right: IN, IN-DNA complexes, K156, K156- DNA complexes. Panel values are the mean ± standard deviation (error bars) of three independent experiments. CRE (cAMP Responsive Element) was used as control. B: Histogram representation of simple ELISA results for the binding of DNAs to MAba4. Panel values are the mean ± standard deviation (error bars) of three independent experiments. C: Histogram representation of an ELISA control for the competitive binding of DNAs to IN and K156 realized in presence of a mouse IgA antibody.

The DNA fragments, LTR 17, 32 and 34 have been designed to adopt monomolecular hairpin structures by folding around a 3-thymine loop (TTT) located at the centre of the oligonucleotides (Fig. 2B). The stems reproduce several versions of the U5 LTR extremity of viral DNA The stem of LTR34 mimics the unprocessed 17 bp version of the LTR extremity, and is sufficient to carry out in vitro integration assays. The stem of LTR32 reproduces the processed version of LTR34, that is a stem with the upper transferred strand lacking 3′ GT (15 nucleotides) and the lower non transferred strand (17 nucleotides including the dangling 5′AC). The shorter LTR version, LTR17 (7 bp), carries the minimum DNA site required for IN binding [1]. The ability of DNA to bind the epitope and impede the formation of the epitope- antibody complex was measured in competition ELISAs (Fig. 4A). The antibody was added to wells coated with IN and K156 either free or incubated with DNA. The strongest inhibition was observed with LTR34 that is an unprocessed version of viral DNA. Control tests showed that LTR oligonucleotides were unable to interact with MAba4 (Fig. 4B) and that the CRE oligonucleotide (Fig. 2B) was unable to interfere in the binding of MAba4 to IN and K156.

All together, above results accredits the idea that the epitope/α4-helix belongs to the DNAbinding domain of the enzyme (Fig. 1A). Implication of the α4-helix into the binding of the enzyme to the LTR extremities has been previously shown by fluorescence anisotropy, circular dichroism, and NMR studies of DNA-peptide complexes [26] and has been confirmed recently by analysis of the crystal structure of PFV IN bound to its cognate DNA [25]. The fact that LTR34 behaves as a better competitor than LTR32 for the formation of the MAba4-K156 complex is conform to our results on the binding of K156 to viral DNA.

### MAba4 inhibits the *in vitro* concerted integration catalyzed by HIV-1 IN

**Figure 5:**
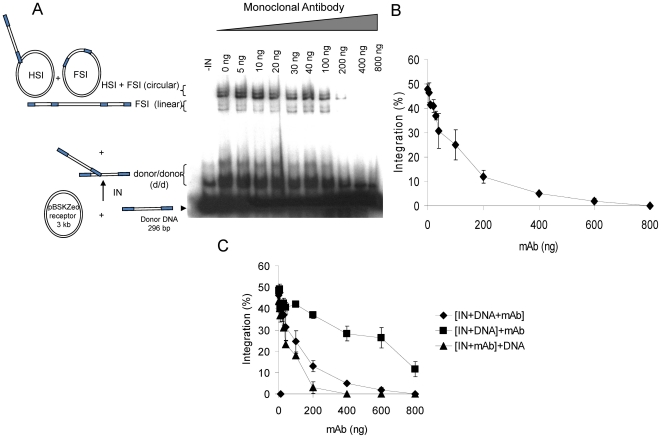
Effect of MAba4 on the HIV-1 IN activity. A: Standard concerted integration assays were performed with 1 pmole of IN in presence of increasing amounts of MAba4. The final NaCl concentration was adjusted to 30 mM. MAba4 was added to the mixture at different concentrations and the reaction products were loaded on a 1% agarose gel: 0 (lane 1), 10 (lane 2), 20 (lane 3), 30 (lane 4), 40 (lane 5), 50 (lane 6), 100 (lane 7), 200 lane (8), 600 (lane 9) or 800 ng (lane 10). The position and the structure of the different products obtained after half-site (HSI), full-site (FSI) and donor/donor integration (d/d) are indicated. B: Densitometry of the FSI (full site integration) and FSI+HIS (half site integration) bands of experiments shown in A. The different integration products were quantified using the Image J software. Panel values are the mean ± standard deviation (error bars) of three independent experiments. C: Inhibition assays were performed under different preincubation conditions. MAba4 was either added simultaneously to IN and DNA ([IN+DNA+MAba4]), either after preincubation between IN and DNA ([IN+DNA]+MAba4) or it was preincubated with IN before adding the DNA substrates ([IN+MAba4]+DNA). The different integration products detected on agarose gel were quantified using the Image J software. Panel values are the mean ± standard deviation (error bars) of three independent experiments.

To assess the inhibitory effect of MAba4 on IN we used the typical *in vitro* concerted integration assay using a processed LTR as a target DNA [49]. Results reported in figures 5A and 5B illustrate the strong inhibition effects of MAba4 on integration this occurring with an apparent IC50  = 150 ng. To learn more on the molecular mechanism underlying the integration inhibition, we also conducted concerted integration assays varying the preincubation conditions. As shown in figure 5C, preincubation of partners plays an important role on the outcome of the results. Thus, when IN is preincubated with MAba4 before adding DNA, inhibition is stronger than when IN is preincubated with DNA before adding MAba4 (IC50<100 ng and IC50>600 ng, respectively). These findings show that MAba4 exerts an important antagonzing effect on the binding of DNA to IN, which is in agreement with our competition ELISA results (Fig. 4).

## Discussion

The use of monoclonal antibodies is proved to be of great help in the search of functional regions in proteins and drug discovery [58], [59], [60], [61]. For most cases, the approach requires a linear immunizing peptide to generate antibodies capable to cross-react with the corresponding segment in the native protein. Literature provides examples of such peptides leading to antibodies recognizing the immunizing peptide and the corresponding segment within the protein with similar efficiency [62], [63], [64]. However, linear peptides are generally structurally unstable compared with segments making often difficult the interpretation of antigen-antibody molecular recognition on a conformational basis. For instance, K159, the linear immunizing peptide used in the present work for preparation of the MAba4 antibody is disordered almost on its whole length, while, in the IN CCD crystal structures, the N-terminal portion (residues 147–166) of the corresponding segment displays a regular helix secondary structure, and the C-terminal portion (166–175) a flexible loop. Despite this, MAba4 recognizes equally well the epitope within the K159 peptide taken in isolation or as a segment within the enzyme. The whole K159 peptide is highly antigenic. It has been previously used to prepare polyclonal antibodies recognizing the flexible C-terminal portion [46]. Once purified up to the stage monospecific the antibodies cross reacted with both IN CCD and IN, and inhibited with high efficiency the IN catalytic activity in *in vitro* integration assays. Since then, several reports have shown that the C-terminal epitope was an optimal epitope of IN (the 164/165–172 sequence) in HIV-1 infected patients of South Africa and Botswana [62]–[65]. In addition to its strong avered immunological properties, the C-terminal portion extensively participates to the binding of LEDGF (lens epithelium derived gross factor) [27], [57], a factor that is thought to tether the preintegration complex to chromatin and favor integration [65], [66], [67]. Moreover, several experiments have suggested its possible involvement in the nuclear transport of IN.

The monoclonal antibody MAba4 obtained in the present work has this time its epitope in the other extremity of K159 that is in the N-terminal portion. Noteworthy, prediction methods have shown that the latter was a functional epitope (“Antibody Epitope Summary” table, from the “HIV-1 molecular immunology database” of Los Alamos; (http://www.hiv.lanl.gov/content/immunology), revealing that experimental results and predictions can be in perfect agreement. However, the C-terminal and the N-terminal portions have completely different conformational properties. While the C-terminal portion is mainly disordered whatever is the context, the N-terminal portion accommodates either an amphipathic helix structure (i.e. in the CCD crystal, thanks to the tertiary structure interactions) or a mainly disordered structure (i.e. in K159, in response to water exposure of amino acid residues). The main question is how MAba4 can bind with an apparent similar efficiency an epitope under both the random coil structure and the helix conformation?

Actually, the N-terminal portion of K159 presents intrinsic α-helix forming properties, as it easily recovers a stable helical conformation in trifluoroethanol, an organic solvent known for its helix enhancer properties [55]. Therefore the N-terminal portion could assume an equilibrium of different conformations with either a low energy barrier between the helix and the other conformations or an entropic effect that only weakly contributes to the α-helix selection. These could explain why in ELISA essays the antibody binds with equal efficiency the epitope either as a stable helix, within IN and K156, or an instable helix, within K159 and pep-a4. This ability of the antibody to select a stable conformation is conform to recent molecular modeling calculations which indicate that a proportion of short antigenic peptides could adopt the desired stable conformations and generate antibodies that in turn will recognize this conformation within the native proteins [64]. It could be also that: the antigenic peptide presents the ability of eliciting a number of conformationally different antibodies each of them recognizing a particular antigenic conformation; or, a single antibody binding site adopts several conformations, each of them capturing a complementary antigen conformation, among a family of preexisting antigen conformations.

Anyway, it appears that the antibody and the DNA have completely different requirements for the binding to IN. The recognition of the epitope by the antibody seems less selective than that of the corresponding peptide by its cognate DNA. In the latter case a pre-stabilization of the peptide in α-helix is required to obtain a specific binding [26].

Finally, the inhibitory properties of the antibody seem them also interesting. Both competition ELISA assays and concerted integration assays under preincubation conditions show that the inhibition mechanism proceeds by means of an impediment of the binding of IN to its cognate DNA. Obviously, these findings reinforce the idea of a significant contribution of the IN α4- helix in the recognition of the viral DNA LTRs.
